# Avoiding drying-artifacts in transmission electron microscopy: Characterizing the size and colloidal state of nanoparticles

**DOI:** 10.1038/srep09793

**Published:** 2015-05-12

**Authors:** Benjamin Michen, Christoph Geers, Dimitri Vanhecke, Carola Endes, Barbara Rothen-Rutishauser, Sandor Balog, Alke Petri-Fink

**Affiliations:** 1 Adolphe Merkle Institute, University of Fribourg, Chemin des Verdiers 4, 1700 Fribourg, Switzerland; 2 Chemistry Department, University of Fribourg, Chemin du Musée 9, 1700 Fribourg, Switzerland

## Abstract

Standard transmission electron microscopy nanoparticle sample preparation generally requires the complete removal of the suspending liquid. Drying often introduces artifacts, which can obscure the state of the dispersion prior to drying and preclude automated image analysis typically used to obtain number-weighted particle size distribution. Here we present a straightforward protocol for prevention of the onset of drying artifacts, thereby allowing the preservation of in-situ colloidal features of nanoparticles during TEM sample preparation. This is achieved by adding a suitable macromolecular agent to the suspension. Both research- and economically-relevant particles with high polydispersity and/or shape anisotropy are easily characterized following our approach (http://bsa.bionanomaterials.ch), which allows for rapid and quantitative classification in terms of dimensionality and size: features that are major targets of European Union recommendations and legislation.

Nanomaterials are present in nearly all segments of modern life, including electronics, cosmetics, food products, and healthcare. In an attempt to ensure the safety of all applications of nanomaterials in products, the European Commission has issued a definition of the term ‘nanomaterial’ to be used in all European Union legislation.^1^ Essentially, a *“nanomaterial [is] a natural, incidental or manufactured material containing particles, in an unbound state or as an aggregate or as an agglomerate and where, for 50 % or more of the particles ****in the number size distribution,**** one or more external dimensions is in the size range 1* *nm – 100* *nm”.* With this definition, the median of the number-weighted size distribution was established as a definitive parameter in the legislation of nanomaterials. Electron microscopy is a so-called counting method, which determines individual nanoparticle size and can be used to construct the required number-weighted size distributions. However, this technique is frequently plagued with issues related to artifacts, statistical reliability and interpretation.^2^ Drying steps, unavoidable during sample preparation, can result in non-uniform particle deposition and particle aggregation.^3^ Characterizing commercially-relevant materials, which often have highly non-uniform sizes and shapes, is particularly challenging. Here we describe a simple and almost universally-applicable approach that can eliminate artifacts found in conventional TEM micrographs taken for the analysis of suspended particulate nanomaterials.

Scanning (SEM) or transmission electron microscopes (TEM) are standard equipment in many companies and research facilities, and their use is continually expanding. Measurements are typically performed in high-vacuum chambers on dry samples. TEM sample preparation typically consists of drop-casting and drying a particle suspension on a TEM grid. This process often results in the formation of nanoparticle aggregates^4^,^5^,^6^ located in segregated patches at the perimeter of the dried droplet, which can be explained by surface dewetting^7^ and the so-called ‘coffee-ring’ effect.^3^ The unambiguous discrimination between ‘true’ aggregates present in the sample and ‘formed’ aggregates, which were produced during sample preparation is very challenging in standard TEM. CryoTEM and liquid-cell TEM are particularly interesting developments that in principle may overcome these issues.^8^,^9^,^10^,^11^,^12^,^13^ However, in practice, these techniques represent a rather expensive class of electron microscopy that require utmost delicacy and are time-consuming. Furthermore, due to the usually limited number of counting events, sample sizes are often not large enough for statistical significance. The method we present here is straightforward, cost-effective, and does not require specialist expertise. By considerably improving the drop-casting deposition pattern – free from ex situ aggregates and clusters – we were able to capture the native, colloidal dispersion state, enabling high-throughput yet accurate quantitative TEM characterization. The efficacy of the technique was confirmed by UV-Vis spectroscopy and light scattering, which both analyze the true unaltered suspension.

The approach relies on the stabilization of individual particles against aggregation, mitigating dewetting, and fortifying Marangoni flow.^14^ All this can be easily achieved in one step by mixing the particle suspension with a dilute solution of a suitable macromolecular agent at the appropriate concentration ratio. Bovine serum albumin (BSA) was chosen as the macromolecular agent. Although BSA is not necessarily the only possible choice, it was chosen as it is cheap, widely available and well-studied.

## Results

### Spherical gold nanoparticles

**Figure 1:**
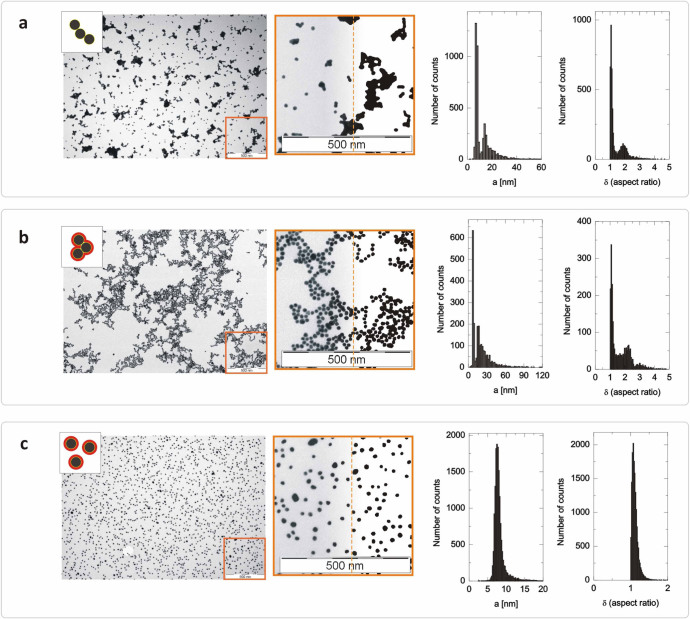
TEM micrographs of drop-cast samples of suspended Au NPs. TEM micrographs of drop-cast samples of suspended Au NPs without BSA (**a**), with BSA but well below the optimal concentration (**b**), and with BSA at the optimal concentration (**c**). The magnified view (middle) - the right side of which is bi-leveled for high contrast - shows aggregation due to van der Waals forces (**a**), aggregation also due to protein bridging (**b**), and stability as single particles owing to the presence of an intact BSA ‘shield’ (**c**).

Model nanoparticles, i.e. gold nanoparticles (Au NPs), were used to introduce and validate the approach before applying it to more challenging and economically important nanopowders (e.g. silica (SiO_2_), titanium dioxide (TiO_2_), zinc oxide (ZnO) and copper carbonate nanoparticles, and highly anisotropic and polydisperse cellulose nanocrystals, t-CNCs). We therefore analyzed TEM micrographs of Au NPs and validated the data independently by UV-Vis spectroscopy and dynamic light scattering (DLS). We distinguished single particles from ex situ aggregates and always compared samples prepared in the standard manner to those prepared using our method, which consisted of mixing an aqueous solution of BSA with the Au NPs, drop casting the suspension onto the TEM grid, and finally letting it dry under ambient conditions. Figure 1 shows TEM micrographs of drop-cast samples of Au NPs suspended without BSA (Figure 1a), with BSA but well below the optimal concentration (Figure 1b), and with BSA at the optimal concentration (Figure 1c). This optimal concentration of the BSA solution (C_0_, mass/vol.) was estimated via equation (1), 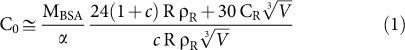
where C_R_ is the mass-based concentration of the particle suspension, 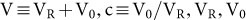 the volume of the particle suspension and BSA solution, respectively, ρ_R_ the mass density of the nanoparticles, R the expected size (e.g. for spheres the radius) of the nanoparticles, M_BSA_ the molar mass of BSA, and α the area occupied by one surface-adsorbed BSA molecule. The derivation of equation (1) is given in the Supplementary Information (SI 1). A rough estimate of the expected particle size is sufficient to prepare the sample and an interactive online platform for calculating the optimal BSA concentration is available: http://bsa.bionanomaterials.ch. In the Supplementary Information, we also provide a straightforward graphical computational tool (SI 1.3, Nomogram) that enables to evaluate equation (1) without the use of e.g. a calculator.

As expected, conventional sample preparation resulted in very densely packed aggregates (Figure 1a), which were accumulated mostly in the final perimeter area of the dried droplet. Even a small amount of BSA (∼C_0_/20) was found to influence the deposition pattern, however, protein bridging^15^,^16^ (i.e. the simultaneous adsorption of the protein onto more than one particle) likely dominated the inter-particle interactions and led to the formation of large clusters (Figure 1b). Individual particles can be easily identified in these clusters even at this low BSA level. Finally, the micrograph of the sample prepared with the optimal amount of BSA (Figure 1c) shows well-distributed single particles without any sign of aggregation. The difference between these drop-cast samples is even clearer when quantifying the analysis of these micrographs. The micrographs were analyzed by fitting ellipses around the particles, following previously reported protocols.^17^,^18^ Each ellipse is defined by two parameters: the long axis (2a) and the aspect ratio (δ ≤ 1), which is calculated as the ratio of the lengths of the long and short axes. The equivalent radius of these spherical particles may be approximated by half the length of the long axis, and particle analyses are summarized in histograms: both a and δ are highly skewed to the right when the sample is overwhelmed with artifacts (Figure 1a and 1b). However, when the Au NPs were prepared with the optimal amount of BSA, the TEM histogram shows a narrow distribution (Figure 1c) with an average radius of 8.1 nm and a polydispersity index of 0.2 (SD / mean). The average aspect ratio is δ = 1.12 with a small dispersion of 0.1 (SD / mean).

### Preserving in situ colloidal dispersion state

**Figure 2:**
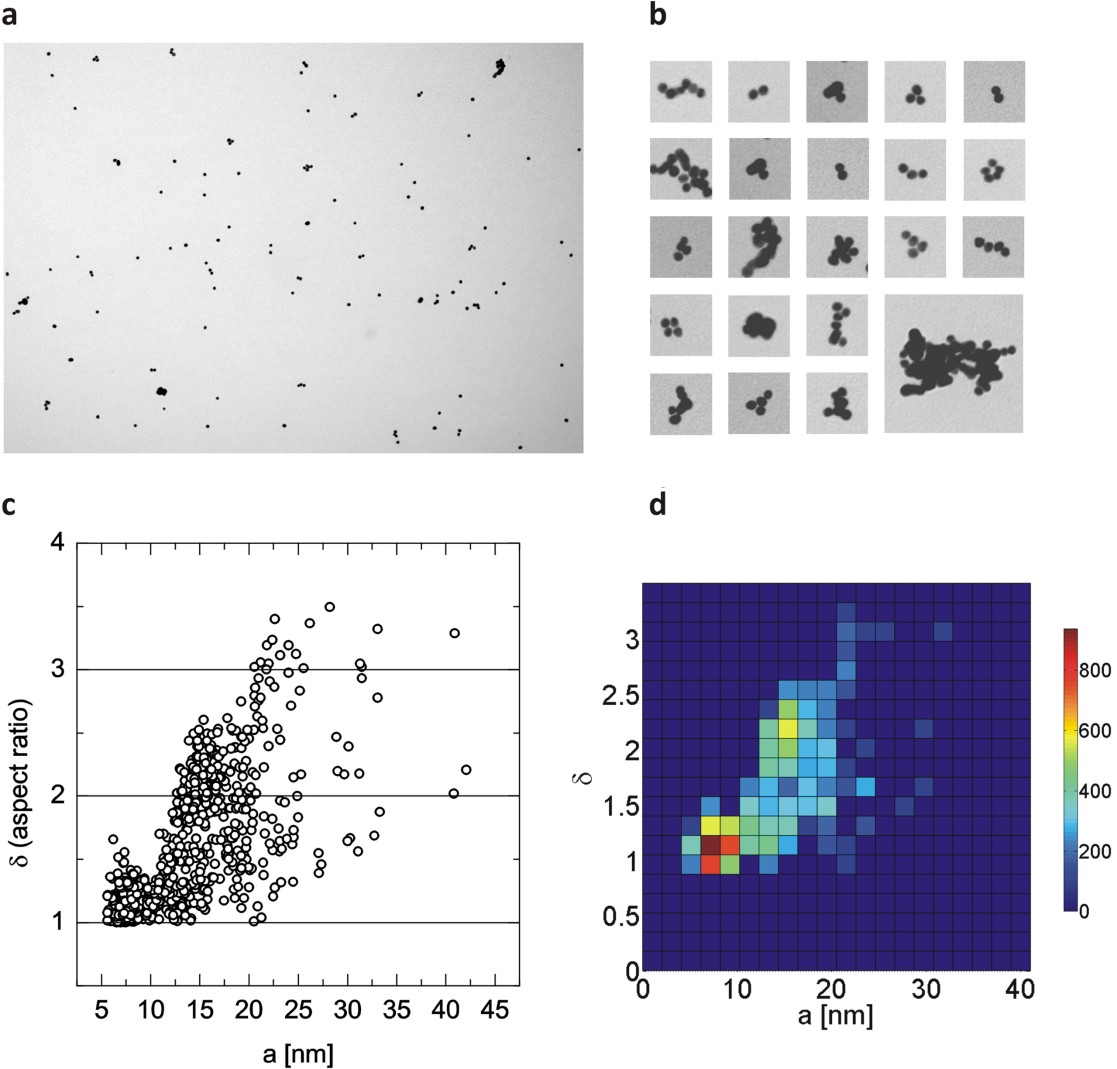
TEM analysis of in situ aggregates of Au NPs. Panel (**a**) and (**b**) depict representative TEM micrographs (width: 2.68 μm) and close-up views of typical in situ aggregates of Au NPs. Panel **c** shows the result of counting 2200 single and pre-aggregated particles. Particles were classified in terms of long axis (2a) and aspect ratio (δ), following the same approach as for Figure 1(a–c). Panel (**d**) depicts the result summarized into a two-dimensional histogram quantifying the occurrence of existing combinations of aspect ratio and size.

In the next step, we increased the complexity of the particle system and Au NPs were deliberately aggregated prior to TEM sample preparation using centrifugation (3500 g for 10 min.). This concentrated suspension was then diluted with a 0.05 wt% citrate solution. Our aim was to create ‘low-degree’ aggregates already present in the suspended state. These are referred to as in situ aggregates in this manuscript. In this case, nontrivial particle morphology can be expected, including single particles next to multiplets. Indeed, this was confirmed by TEM. The micrographs clearly show that particle doublets, triplets, quartets, etc. were present in addition to single particles (Figure 2a and 2b). As anticipated, larger aggregates showed arbitrary morphologies: between chain-like oligomers and close-packed globules several shapes and types of packing were observed. Analysis of TEM micrographs revealed that the dimensionality and size of these in situ aggregates varied over a wide range (Figure 2c). The occurrence of existing combinations of aspect ratio and size can be summarized in a two-dimensional joint histogram (Figure 2d). Such information allows rapid and quantitative classification in terms of dimensionality and size: features that are in the focus of EU recommendations and legislation.^1^,^19^

**Figure 3:**
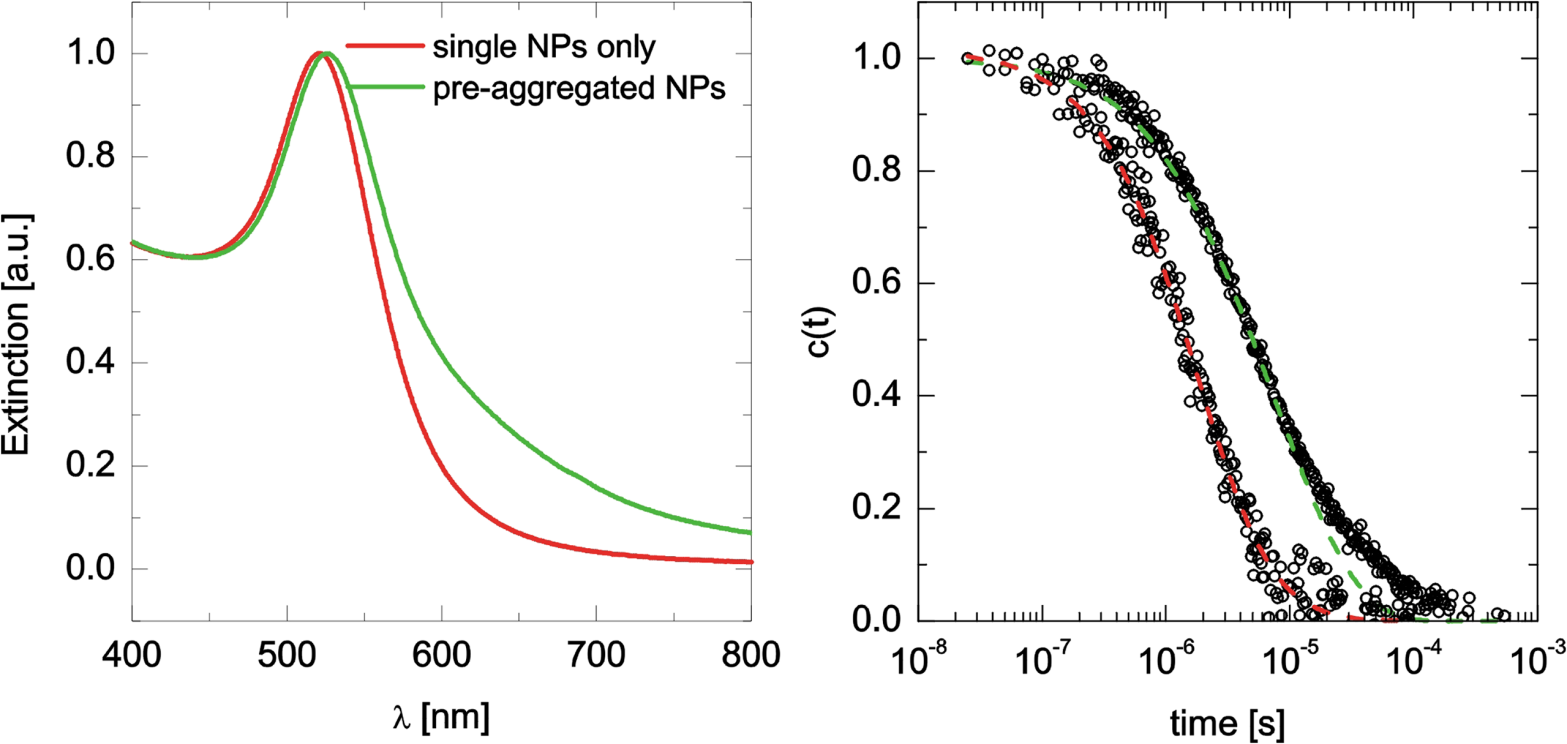
In situ characterization of Au NPs. UV-Vis extinction spectra (left) and dynamic depolarized light scattering results (right, empty circles) of single and in situ aggregated NPs. The dashed lines are correlation functions reconstructed from TEM analysis of BSA-prepared samples (Supplementary Information, SI 2).

To confirm that sample preparation using BSA did preserve the in situ colloidal dispersion state in terms of size and dimensionality, both the single and in situ aggregated Au NPs were analyzed also in their suspended native state using UV-Vis and DLS (Figure 3). These truly in situ measurements were then compared to the TEM results of samples prepared with the optimal concentration of BSA. For single Au NPs, the particle size estimated from the UV-Vis spectrum (Figure 3), in which the center position of the localized surface plasmon resonance is at λ_c_ = 520.7 nm, indicating a particle radius of 8–9 nm,^20^ agrees very well with the result obtained from TEM. The UV-Vis spectrum of in situ aggregated Au NPs displayed the signatures of predominantly low-degree aggregates: a clear red-shift of the center position as well as an increase in the extinction towards longer wavelengths. DLS provided further proof of the preserved suspended state. Based on particles observed and counted in TEM micrographs, correlation functions were reconstructed (Figure 3, right side, dashed lines) and plotted over correlation functions recorded from actual suspensions of single and in situ aggregated NPs prior to drop casting. The overall agreement between the results of these considerably distinct approaches is excellent. The reconstruction of correlation functions from TEM analysis is explained in detail in the Supplementary Information (SI 2).

### Real-life nanomaterials

**Figure 4:**
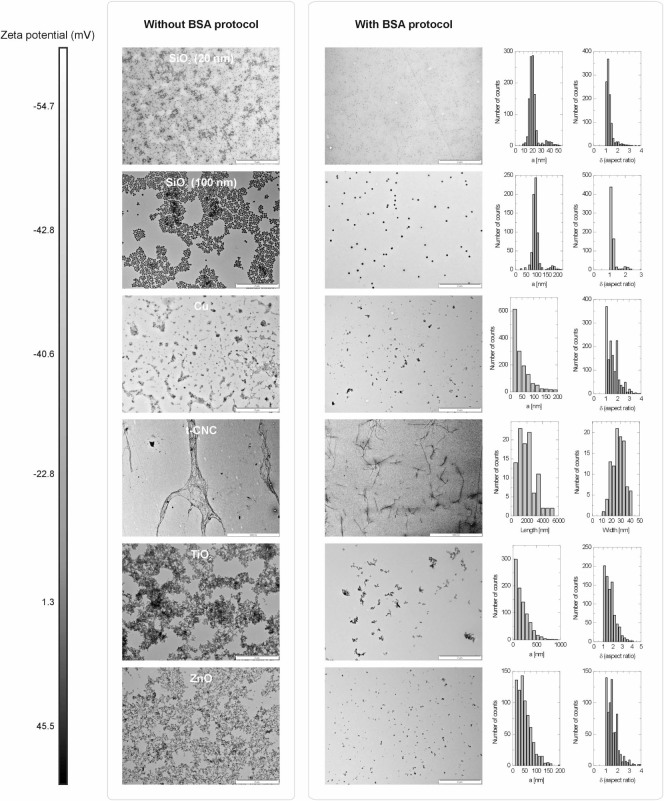
Micrographs of various suspended nanopowders deposited onto the TEM grid without and with BSA. The result of image analysis is summarized by the histograms. The Zeta potential, relevant for describing the effective surface charge, increases from top to bottom.

We then selected five representative particulate nanomaterials to test the efficacy of our method. We chose nanopowders which are economically relevant, produced on large scales, or which show large polydispersity and/or shape anisotropy, ensuring that a large zeta potential range was covered (from −55 mV to +46 mV). As such, we investigated SiO_2_, Cu, TiO_2_ and ZnO nanoparticles, in addition to cellulose nanocrystals derived from tunicate styela clava (t-CNCs).^21^,^22^ Sample preparation generally followed the protocol described above for the Au NPs. Non-spherical particles (e.g. ZnO) required a slight adaptation of the method (Supplementary Information SI 1.1). TEM micrographs showed that our concept of sample preparation is also applicable to much more complex particulate nanomaterials (Figure 4). The micrographs of the samples without BSA show highly aggregated structures on the TEM grids for all of the various nanomaterials. Utilizing BSA, however, enabled automated image analysis of the nanoparticles. Even cellulose nanocrystals, which are the most complex materials used in this study and whose TEM micrographs are strongly affected during the drying process in the absence of BSA, become accessible for quantitative analysis. TEM proved to be particularly relevant for this material since in situ characterization (e.g. light scattering) of CNCs is very challenging due to the fiber-like shape of cellulose nanocrystals and their considerable polydispersity in both length and width. The developed BSA protocol was also found to be successful over the entire zeta potential range. In particular, the neutral and positively charged particles (TiO_2_, ZnO) show extremely good results with BSA, as their aggregation and accumulation upon drying without BSA makes the resulting micrographs almost impossible to analyze via standard TEM. Using DLS, these samples were also analyzed in their suspended native state and the result was compared to that of TEM. Based on particles observed and counted in TEM micrographs, correlation functions were reconstructed and plotted over correlation functions recorded from the suspensions prior to drop casting (Supplementary Information, Figure SI 6). Similarly to the case of Au NPs, the results of these two distinct techniques agree very well.

### Particle deposition and BSA coating

**Figure 5:**
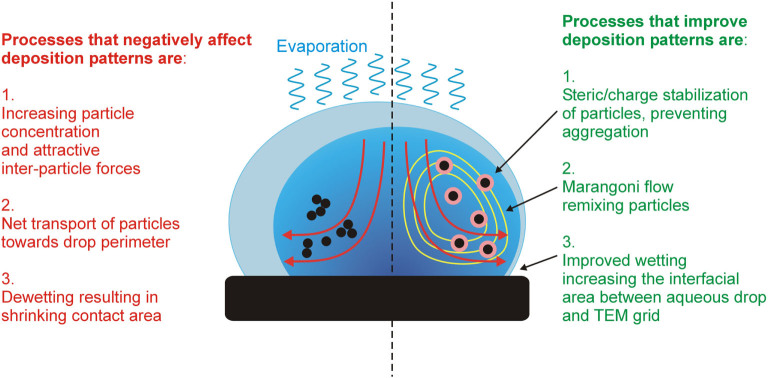
Illustration of an aqueous drop and the dominant processes influencing the deposition pattern of suspended particles onto a hydrophobic surface. Red arrows indicate effective particle flow due to the coffee ring effect. Yellow lines show counter flow introduced by the Marangoni flow.

Figure 5 illustrates an aqueous drop and the dominant processes influencing the deposition pattern of suspended particles onto a hydrophobic surface. Increasing particle concentration, attractive inter-particle forces and a dewetting grid surface negatively affect the deposition pattern of the particles on the TEM grids. The presence of the right concentration of BSA strongly influences these three parameters, resulting in drop-cast samples of good quality. Attractive inter-particle forces lead to aggregation,^4^,^5^,^6^ which is enhanced by drying as the particle concentration increases.^23^ Yet, aggregation can be prevented by stabilizing the colloidal particles, deploying effects such as steric hindrance or surface charge. BSA, if covering the entire surface of the particles, promotes stability, since the protein adsorbed onto the surface has been shown to form a thin protective shell^24^,^25^,^26^ that shields the van der Waals interactions between particles.^27^ Thus, even as the particle concentration continuously increases during drying, the protein shell maintains the colloidal stability and prevents aggregation. The overall number of particles and corresponding surface area can be easily estimated via the mass-based concentration of the particle suspension, and thus the number of BSA molecules required to form a monolayer around each particle can be calculated (Supplementary Information, S1). Monolayer coverage was based on our findings that show that at least one complete layer of BSA is required to prevent nanoparticles from aggregation induced by polymer bridging (Figure 1b). This was supported by previous studies, which have shown that BSA can form a regular monolayer on citrate-stabilized gold NPs via an electrostatic mechanism.^28^,^29^ Electrostatic interactions are the primary driving force for the adsorption, and it has been previously shown that protein conformation and charge distribution guide the protein-particle interaction.^38^ As a consequence, NPs can interact with oppositely charged regions in a protein, even if both the NPs and the proteins display a net positive or net negative charge.^38^

The model can be easily generalized for non-spherical as well as polydisperse particles. For a given particle mass, any deviation from the spherical shape increases the surface area, and for any given shape, this increased surface area can be estimated. However, calculating the surface area of highly irregular particles is not trivial. As an alternative, we propose to add more BSA than what was calculated for spheres with smooth surfaces. This simplistic approach was tested using gold NPs (15 nm) and SiO_2_ NPs (100 nm), and it was found that up to an eightfold increase of the BSA concentration no negative influence (with respect to image quality) was observed on the micrograph. Beyond this value, BSA stains were clearly visible in the TEM micrographs, yet the sample deposition remained good (Supplementary Information, SI 1.1). Analysing the statistical moments of the size distribution, it can be shown that polydispersity decreases the overall surface area and is thus not a particularly critical parameter for sample preparation (Supplementary Information, SI 1.2). TEM grids are frequently coated with a hydrophobic polymer film such as poly(vinyl formal) resin. Accordingly, the interface between the substrate and the aqueous droplet favors a minimal contact area (Supplementary Information, SI 4, video file: “Control.mov”).^7^ Consequently, a non-negligible area of the TEM grid remains vacant and suspended particles are not deposited uniformly but only found in segregated areas. However, if the surface tension is sufficiently reduced, the contact area between the drop and the substrate can remain constant during drying (Supplementary Information, SI 4, video file: “BSA.mov”). As the evaporation rate is not constant along the surface of the drop, capillary flows are induced within the drying drop, resulting in the aforementioned coffee-ring effect.^3^ This convective capillary flow carries the particles towards the periphery of the drying droplet, and consequently, the particles accumulate near the drop perimeter. It has been previously shown that small particles tend to accumulate closer to the periphery than larger ones.^30^ Surface tension can induce a thermo-capillary flow referred to as the Marangoni flow.^14^ Capillary forces can be influenced for either suppressing or promoting self-assembly of particles in a controlled manner.^31^,^32^ In highly volatile solvents this flow can reduce and even neutralize the ‘coffee-ring’ effect.^33^ However, temperature-driven Marangoni flow does not readily build up in aqueous suspensions^34^, but can be strengthened by the presence of a surfactant creating a gradient in the surface tension, which strengthens the Marangoni flow.^35^,^36^,^37^ BSA exhibits hydrophobic and hydrophilic regions^38^ and similarly to surfactants, BSA is able to reduce the surface tension at the air-liquid interface of the aqueous drop.^39^ This lowers the surface tension and thus improves the wetting of the substrate. Due to the convective capillary flows resulting in the ‘coffee-ring’, the concentration of a surfactant will not be uniform along the air-liquid interface: the concentration is the highest at the ‘pinned’ perimeter and gradually decreases with height. This surface-tension gradient induces a convective flow moving from regions of low to high surface tension, which stirs the fluid, removes the particles from the periphery, and carries them towards the center of the drop. This remixing improves the homogeneity of the drying suspension and promotes the uniformity of the particle deposition.

## Conclusion

We have introduced a simple protocol for prevention of the onset of drying artifacts in drop-cast TEM samples, and have demonstrated how to prevent decoupling of the ex situ TEM analysis from the in situ dispersion state. This is of paramount importance for users of conventional TEM in academic research and industry, since it allows the preservation of in situ colloidal features (size, morphology, and dimensionality) during sample preparation by adsorption of a thin protein layer onto the particle surface. Aside from electrosteric stabilization, the effect of BSA on the surface tension of a drying droplet leads to uniform particle deposition and enables high-throughput quantitative characterization of a wide range of suspended nanomaterials.

## Methods

Au NPs were prepared by reduction of gold(III) with citrate (molar ratio of citrate to Au = 3), following the Turkevich-Frens method.^40^,^41^ Sodium citrate tribasic dehydrate and gold(III) chloride trihydrate were purchased from Sigma-Aldrich and Fluka, respectively. The large SiO_2 _nanoparticles were synthesized via the Stöber method.^42^ The NPs are formed by an ammonia-catalyzed condensation reaction of hydrolyzed tetraethylorthosilicate (TEOS). 58 mL deionized water and 7.8 mL NH_3_ were added to 162 mL ethanol and the mixture heated to 65 °C. Under strong stirring, 22 mL of preheated TEOS was added to the ethanol/ammonia/water mixture. Finally, the nanoparticles were centrifuged at 10,000 g and washed six times with MilliQ water. The small SiO_2 _nanoparticles were synthesized by the procedure of Hartlen.^43^ The nanoparticles were synthesized in water, containing L-arginine as catalyst, by adding TEOS to a cyclohexane organic layer. TEOS slowly migrated into the aqueous phase where it hydrolyzed and condensed. 9.1 mg of L-Arginine were dissolved in 6.9 mL MilliQ water, to which then 0.45 mL of cyclohexane was added. While stirring gently, this solution was heated to 63 °C. After temperature stabilization, 0.55 mL TEOS was added slowly to the top organic layer, which was followed by stirring for 24 hours. The cyclohexane was then allowed to evaporate and the solution was dialyzed against MilliQ water. TiO_2 _nanoparticles were obtained from Degussa P-25 powder (Sigma-Aldrich). The powder, as received, was dispersed in water, sonicated for ten minutes and diluted to the desired concentration. The copper carbonate nanoparticles (Cu) were obtained from a commercially available sample used for wood impregnation, and were used as received, without any modification. For reasons of confidentiality and trade secrecy, the detailed content and synthetic route was not disclosed. ZnO nanoparticles were obtained in powder form (Sigma-Aldrich 721077). The powder, as received, was dispersed in water, sonicated for ten minutes and diluted to the desired concentration. BSA lyophilized powder, with fatty acid and globulin content smaller than 1%, was purchased from Sigma-Aldrich. BSA solutions were prepared by dissolving the powder in Milli-Q water, followed by ten minutes of sonication and filtration through a 0.2 µm low-protein-binding syringe filter. The particle suspensions were diluted to the desired concentration using MilliQ water. The corresponding BSA solutions were diluted from a ‘standard’ solution of 1.5 mg/mL (15 mg BSA dissolved in 10 mL MilliQ water and sonicated for ten minutes). 100 µL particle dispersion and 100 µL of corresponding BSA solution were mixed and stored at 4 °C prior to drop casting. The Formvar-coated 200 mesh copper grids were placed onto parafilm, and the overall volume of the drop cast was chosen to be V = 5 μL (c = 1). This droplet volume resulted in a perimeter with a radius of approximately 1.34 mm, and thus, the corresponding area of the TEM grid was F ≈ 5.6 mm^2^. We chose V_0_ and V_R_ to be equal, and obtained C_0_ via equation (1). The drop dried for over 2 hours under a fume hood before TEM imaging. Electron micrographs of the Au NPs were taken with a FEI Morgagni and a FEI/Philips CM-100 Biotwin transmission electron microscope (FEI, Hillsboro, Oregon, USA) operating at 80 kV. 16-bit bitmap images were recorded onto a CCD sensor of 970 times 2100 pixels with an image resolution of 1.112 pixel/nm (Morada, Olympus SIS, Germany). The images were bi-leveled in ImageJ (National Institutes of Health NIH, USA) using the default threshold method: IsoData-based variation.^44^ To measure the particles, a built-in routine (ImageJ, Analyze Particles) was used, without separation methods and constrains. The effective surface charge of the nanoparticles was characterized at 25 °C, using phase amplitude light scattering (Brookhaven, ZetaPALS), and the Zeta potential was estimated via the Henry equation.^45^ UV-Vis extinction spectra of the suspended single and in-situ-aggregated Au NPs were recorded at 25 °C using a Jasco V-670 spectrophotometer, using 10-mm-path-length quartz cuvettes. Light scattering measurements were performed at constant temperature (21 °C) using a commercial goniometer instrument (3D LS Spectrometer, LS Instruments AG, Switzerland). The primary beam was formed by a linearly polarized and collimated laser beam (HeNe, 632.8 nm, 21 mW), and the scattered light was collected by single-mode optical fibers equipped with integrated collimation optics. The collected light was coupled into two high-sensitivity APD detectors (Perkin Elmer, Single Photon Counting Module), and their outputs were fed into a two-channel multiple-tau correlator (Correlator.com). The signal-to-noise ratio was improved by cross-correlating these two channels. Videos of aqueous droplets drying on the TEM grid were recorded with a digital camera (Olympus DP72) mounted onto a stereomicroscope (Olympus SZX16). By means of a mirror, both the top and side views of the TEM grid were simultaneously recorded.

## Author Contributions

B. M. conceived the project and carried out the fundamental proof of concept with gold nanoparticles, C. G. executed the experiments with all other nanoparticles. S.B. derived the theoretical treatment for polydispersity and shape and analyzed DLS data. D. V. was creating the nomograms and was strongly involved in all TEM discussions, C. E. in part carried out CNC experiments and TEM imaging thereof. S. B., B. R and A. F. wrote the manuscript and all authors contributed to critical discussion of the subject.

## Supplementary Material

Supplementary Information Supplementary Information
