# Correction: Avoiding drying-artifacts in transmission electron microscopy: Characterizing the size and colloidal state of nanoparticles

**DOI:** 10.1038/s41598-025-33795-w

**Published:** 2025-12-30

**Authors:** Benjamin Michen, Christoph Geers, Dimitri Vanhecke, Carola Endes, Barbara Rothen-Rutishauser, Sandor Balog, Alke Petri-Fink

**Affiliations:** 1 Adolphe Merkle Institute, University of Fribourg, Chemin Des Verdiers 4, 1700 Fribourg, Switzerland; 2 Chemistry Department, University of Fribourg, Chemin du Muse’e 9, 1700 Fribourg, Switzerland

Correction to: *Scientific Reports* 10.1038/srep09793, published online 12 May 2015

This Article contains errors. The Supplementary Information Video file, which was included with the initial submission, was omitted from the original version of this Article. The Supplementary Information is now linked to this correction notice.

## Supplementary Information

Supplementary Video 1.

Supplementary Video 2.

