# Supplementary material for: Avoiding drying-artifacts in transmission electron microscopy: Characterizing the size and colloidal state of nanoparticles
Source: Sci Rep. 2015 May 12;5:9793. doi: 10.1038/srep09793 (PMC4428270; doi:10.1038/srep09793)
Supplement: Supplementary Information [file srep09793-s1.docx]

**Supplementary Information**

Avoiding drying-artifacts in transmission electron microscopy: Characterizing the size and colloidal state of nanoparticles

Benjamin Michen^a†^, Christoph Geers^a†^, Dimitri Vanhecke^a,^ Carola Endes^a^, Barbara Rothen-Rutishauser^a^, Sandor Balog^a^*, Alke Petri-Fink^a,b^*

^a^ Adolphe Merkle Institute, University of Fribourg, Chemin des Verdiers 4, 1700 Fribourg, Switzerland

^b^ Chemistry Department, University of Fribourg, Chemin du Musée 9, 1700 Fribourg, Switzerland

^†^ These authors contributed equally

Contact authors: [sandor.balog@unifr.ch](mailto:sandor.balog@unifr.ch); [alke.fink@unifr.ch](mailto:alke.fink@unifr.ch)

**1. Calculation of BSA concentration for sample preparation**

Let the particle radius be $R$ (Figure S1), and the mass-based concentration of the particle suspension be $C_{R}$. Then the total mass in a droplet with a volume of $V_{R}$ is

$m_{R}=V_{R}C_{R}$. (1.1)

Accordingly, the overall number of particles is

$N_{R}=\frac{m_{R}}{\rho_{R}\frac{4}{3}\pi R^{3}}$, (1.2)

where $\rho_{R}$is the mass density of the particles. The overall surface of the particles is

${A_{R}=N}_{R} 4\pi R^{2}$. (1.3)

The number of BSA molecules needed to form a monolayer around the particles is

$N_{1}=\frac{1}{\alpha}A_{R}$=$\frac{1}{\alpha}\frac{3}{\rho_{R}}\frac{V_{R}C_{R}}{R}$ , (1.4)

where $\alpha$ is equal to the surface area that a single BSA molecule covers. BSA protein adsorbed to gold surface is reported to cover an average surface area of approximately $\alpha=27$ nm^2^.[^1^](#_ENREF_1)^,^[^2^](#_ENREF_2) Let the BSA solution added to the particle suspension be described by its overall volume $V_{0}$ and mass-based BSA concentration $C_{0}$. Accordingly:

$m_{\mathrm{BSA}}=V_{0}C_{0}$ , (1.5)

and the overall number of BSA molecules is

$N=\frac{m_{\mathrm{BSA}}}{M_{\mathrm{BSA}}} N_{A}$ , (1.6)

where $M_{\mathrm{BSA}}\cong66$ kg/mol is the molar mass of BSA, and the mass of one single BSA molecule is $\frac{M_{\mathrm{BSA}}}{N_{A}} (N_{A}\cong6.022 {10}^{23} \mathrm{mol}^{-1})$.


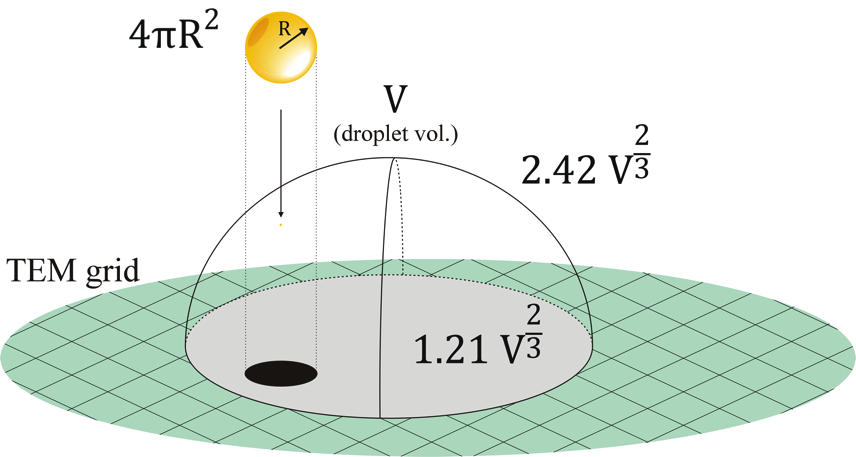


**Figure S1**. Illustration of the dimensions relevant for calculating the particle and BSA concentrations for TEM sample preparation.

Let the overall volume of the liquid drop cast onto the TEM grid be $V=V_{0}+ V_{R}$. The shape and the area of the air-liquid interface of the corresponding drop are non-trivial functions of the drop volume and the surface tension coefficients.[^3^](#_ENREF_3)^,^[^4^](#_ENREF_4) However, if the drop is sufficiently small and the contact angle is not larger than 90 deg., the initial shape can be well approximated by a regular hemisphere.[^5^](#_ENREF_5) The initial drop volume defines the grid area ($F$) that will be covered by the drop. If the initial shape of the drop is approximately a regular hemisphere, this area is $F\cong1.21 V^{\frac{2}{3}}$, and the area of the air-liquid interface is

$A=2\pi\left( \frac{3V}{4\pi} \right)^{\frac{2}{3}}\cong2.42 V^{\frac{2}{3}}$ . (1.7)

The number of BSA molecules needed to cover this area is

$N_{2}=\frac{1}{\alpha}A$. (1.8)

Further BSA molecules are needed for inducing the Marangoni flow driven by the gradient of the surface tension. Based on a series of systematic experimental trials, we found that the coffee ring effect can be successfully prevented if the number of these excess molecules is

$N_{3}\geq9 (N_{2}+N_{1})$. (1.9)

It follows from equation 1.1-9 that

$N_{1}+N_{2}+N_{3}=N$. (1.10)

By expanding and rearranging equation 1.10, we obtain

$C_{0}=\frac{M_{\mathrm{BSA}}}{N_{A}\alpha} \frac{5 (6 V_{R}C_{R}+6^{2/3}\pi^{1/3} R \rho_{R}{({V_{R}+V}_{0})}^{2/3})}{R \rho_{R} V_{0}}$. (1.11)

If we define

$c\equiv\frac{V_{0}}{V_{R}}$ (1.12)

then

$C_{0}\cong\frac{M_{\mathrm{BSA}}}{N_{A} \alpha} \frac{24 \left( 1\text{ }+ c \right) R \rho_{R} + 30 C_{R}\sqrt[3]{V}}{c R \rho_{R} \sqrt[3]{V}}$. (1.13)

**1.1 Non-spherical particles:** At given particle mass, any deviation from the spherical shape of a smooth surface will increase the surface area. Therefore, the expressions corresponding to the volume and surface spherical NPs in equation 1.2 and 1.3, respectively, are to be changed. If the shape is known and is regular, the increased surface area can be easily estimated and accounted for. To do so, we define the shape factor ($S_{F}$), which is the ratio given by the surface of the actual particle shape over that of the spherical shape of equivalent volume. For example, if the NPs are cubes, their surface is $1.24$ times larger than spherical NPs of the same volume, and thus, $S_{F}=1.24$. Accordingly, equation 1.3 and 1.13 modifies: ${A_{R}=N}_{R} 4\pi R^{2} S_{F}$ and $C_{0}\cong\frac{M_{\mathrm{BSA}}}{N_{A} \alpha} \frac{24 \left( 1\text{ }+ c \right) R \rho_{R} + 30 C_{R} S_{F} \sqrt[3]{V}}{c R \rho_{R} \sqrt[3]{V}}$. (1.14)

This approach can be extended to describe other well-defined shapes. Powders, among others, however, are of arbitrary shape and size. In general, they possess rather complex morphologies, which are irregular and varies from particle to particle, and to calculate the overall surface area is not trivial. As an alternative, we propose another approach overcoming this very problem: one may add more BSA than what is calculated for spheres with smooth surfaces. This approach was tested, using gold NPs (15 nm) and SiO_2_ NPs (100 nm) and it was found that up to an eightfold increase of the concentration of BSA no negative influence was observed on the micrograph. Beyond this value, BSA stains were clearly visible in the TEM micrograph, yet the sample deposition remained good (Figure S2).


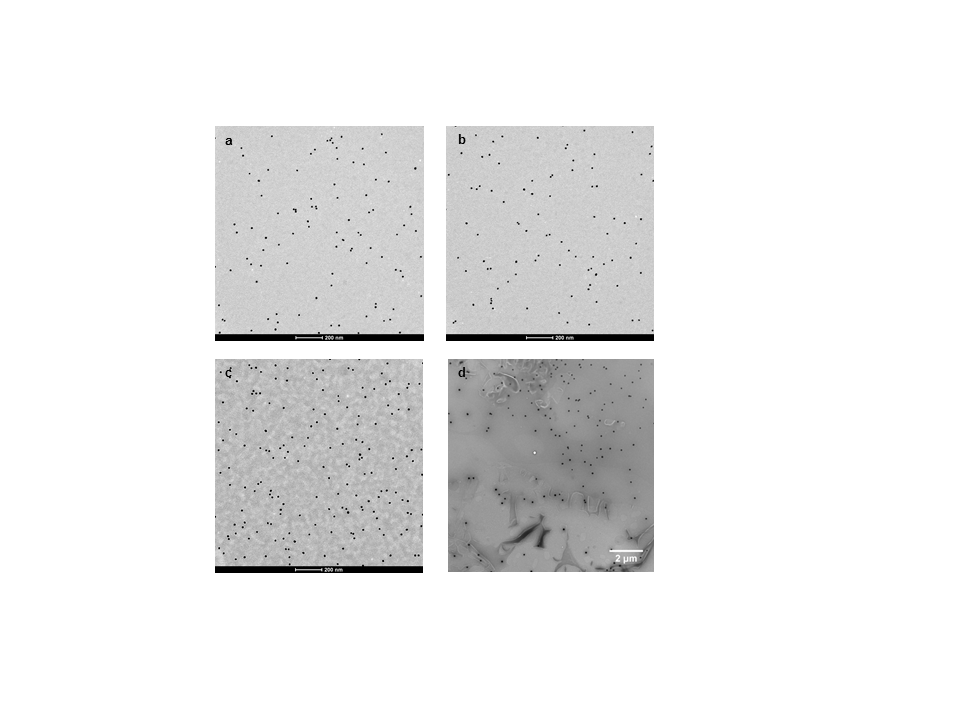


**Figure S2**. TEM micrographs of gold NPs (15 nm) with 2x (a), 4x (b) and 8x (c) higher BSA concentration than the optimal amount calculated. A TEM micrograph of silica NPs (100 nm) with 8x higher BSA concentration than the optimal amount is shown in panel d.

**1.2 Polydisperse particles:** In case of polydisperse spherical particles, equation 1.1 can be written as

${V_{R}C_{R}=m}_{R}=\sum_{i=1}^{n} m_{i}=\rho_{R}\sum_{i=1}^{n} V_{i}=\rho_{R}\frac{4}{3}\pi\sum_{i=1}^{n} R_{i}^{3}.$ (1.15)

$n$ is the total number of polydisperse particles, $m_{i}$ the mass, $V_{i}$the volume, and $R_{i}$ the radius of the $i$^th^ particle. The term $\sum_{i=1}^{n} R_{i}^{3}$can be written as $n\frac{1}{n}\sum_{i=1}^{n} R_{i}^{3}$

where

$\left\langle R^{3} \right\rangle\equiv\frac{1}{n}\sum_{i=1}^{n} R_{i}^{3}$ (1.16)

is equal to the third moment of the particle size distribution. Hence, equation 1.2 is now written as

$n=\frac{m_{R}}{\rho_{R}\frac{4}{3}\pi\left\langle R^{3} \right\rangle}$ (1.17)

Equation 1.3 modifies similarly, and the total surface of the particles is

$A_{R}=\sum_{i=1}^{n} A_{i}=4\pi\sum_{i=1}^{n} R_{i}^{2}=n\frac{1}{n}\sum_{i=1}^{n} R_{i}^{2}= n 4\pi\left\langle R^{2} \right\rangle$ (1.17)

where $A_{i}$ is the surface of the $i$^th^ particle, and

$\left\langle R^{2} \right\rangle\equiv\frac{1}{n}\sum_{i=1}^{n} R_{i}^{2}$ (1.18)

is equal to the second moment of the particle size distribution. We note that the standard deviation of the distribution is equal to $\sigma=\sqrt{\left\langle R^{2} \right\rangle-\left\langle R \right\rangle^{2}}$, where

$\left\langle R \right\rangle\equiv\frac{1}{n}\sum_{i=1}^{n} R$ (1.19)

is the average radius. For polydisperse particles, equation 1.4 is written as

$N_{1}=\frac{1}{\alpha}A_{R}=\frac{1}{\alpha}n 4\pi\left\langle R^{2} \right\rangle=\frac{1}{\alpha}\frac{m_{R}}{\rho_{R}\frac{4}{3}\pi\left\langle R^{3} \right\rangle}4\pi\left\langle R^{2} \right\rangle=\frac{1}{\alpha} \frac{3 V_{R}C_{R}}{\rho_{R}} \frac{\left\langle R^{2} \right\rangle}{\left\langle R^{3} \right\rangle}$. (1.20)

In case of uniform particles $\frac{\left\langle R^{2} \right\rangle}{\left\langle R^{3} \right\rangle}$ = 1, while in case of polydispersity the exact value of $\frac{\left\langle R^{2} \right\rangle}{\left\langle R^{3} \right\rangle}$ depends on the distribution itself. Here we restrict our calculation to normally distributed values, which is likely the most known distribution of all. (Naturally, the value can be calculated for other distributions as well.) The normal distribution is described by two parameters: mean ($\left\langle R \right\rangle$) and standard deviation ($\sigma$):

$P\left( R \right)\equiv\frac{1}{\sqrt{2\pi} \sigma}ⅇ^{-\frac{{(R-\left\langle R \right\rangle)}^{2}}{2\sigma^{2}}}$. (1.21)

The mean, second and third moments are

$\left\langle R \right\rangle=\int R P\left( R \right)\mathrm{dR}$

$\left\langle R^{2} \right\rangle=\int R^{2} P\left( R \right)\mathrm{dR}$

$\left\langle R^{3} \right\rangle=\int R^{3} P\left( R \right)\mathrm{dR}$, (1.22)

and one obtains

$\frac{\left\langle R^{2} \right\rangle}{\left\langle R^{3} \right\rangle}= \frac{\left\langle R \right\rangle^{2}+\sigma^{2}}{\left\langle R \right\rangle^{3}+3\left\langle R \right\rangle\sigma^{2}}$ . (1.23)

Polydisperse particles are usually described by their so-called polydispersity index ($\gamma$) defined as the standard deviation over the mean: $\gamma\equiv\sigma/\left\langle R \right\rangle$. By substituting the definition into equation 1.23 we find

$\frac{\left\langle R^{2} \right\rangle}{\left\langle R^{3} \right\rangle}=\frac{1}{\left\langle R \right\rangle} \frac{1+\gamma^{2}}{1+3\gamma^{2}}$. (1.24)

Finally, we obtain

$N_{1}=\frac{1}{\alpha} \frac{3 V_{R}C_{R}}{\rho_{R}} \frac{1}{\left\langle R \right\rangle} \frac{1+\gamma^{2}}{1+3\gamma^{2}}$ . (1.25)

Figure S3 displays the values of $f\left( \gamma\right)\equiv\frac{1+\gamma^{2}}{1+3\gamma^{2}}$ on the range of $0\leq\gamma\leq1$. It is found that size polydispersity, actually decreases the overall surface area of the particles, and thus less BSA molecule is needed to form a monolayer.


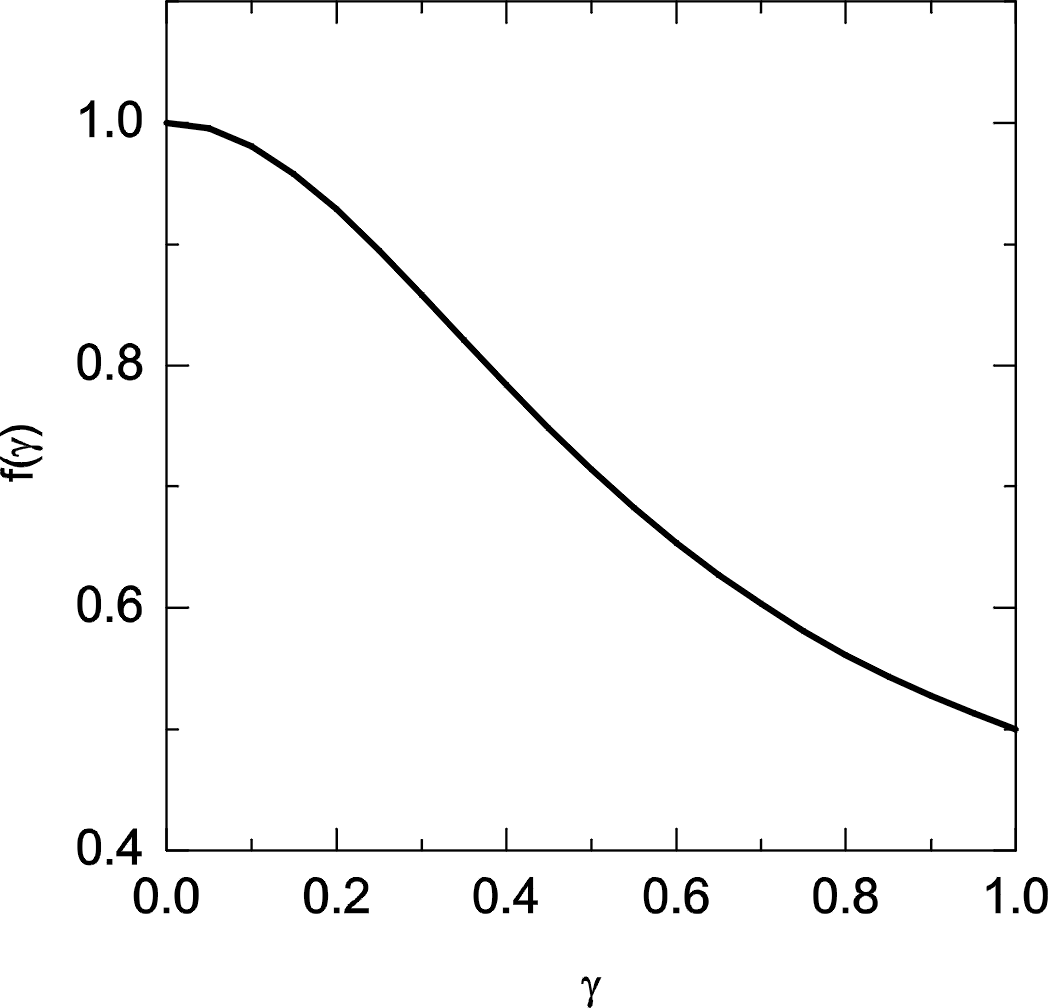


**Figure S3**: The influence of size polydispersity on the overall surface area of the particles.

**1.3 Nomogram**

The nomogram is a graphical computational tool that enables to calculate the optimal BSA concentration without the use of e.g. a calculator. The nomograms presented here are designed for an overall drop volume of $V=5 \mu L$, where the volume of the NPs suspension is equal to that of the BSA solution: $V_{R}=V_{0}$ and $c=1$. We designed two monograms: one for smaller (2-20 nm) and one for larger (20-100 nm) NPs.

The computation, which we demonstrate with SiO_2_ particles, is performed in three steps (Figure S4). Let the concentration of the NPs suspension be 250 µg/mL, and the expected size be not smaller than 30 nm.


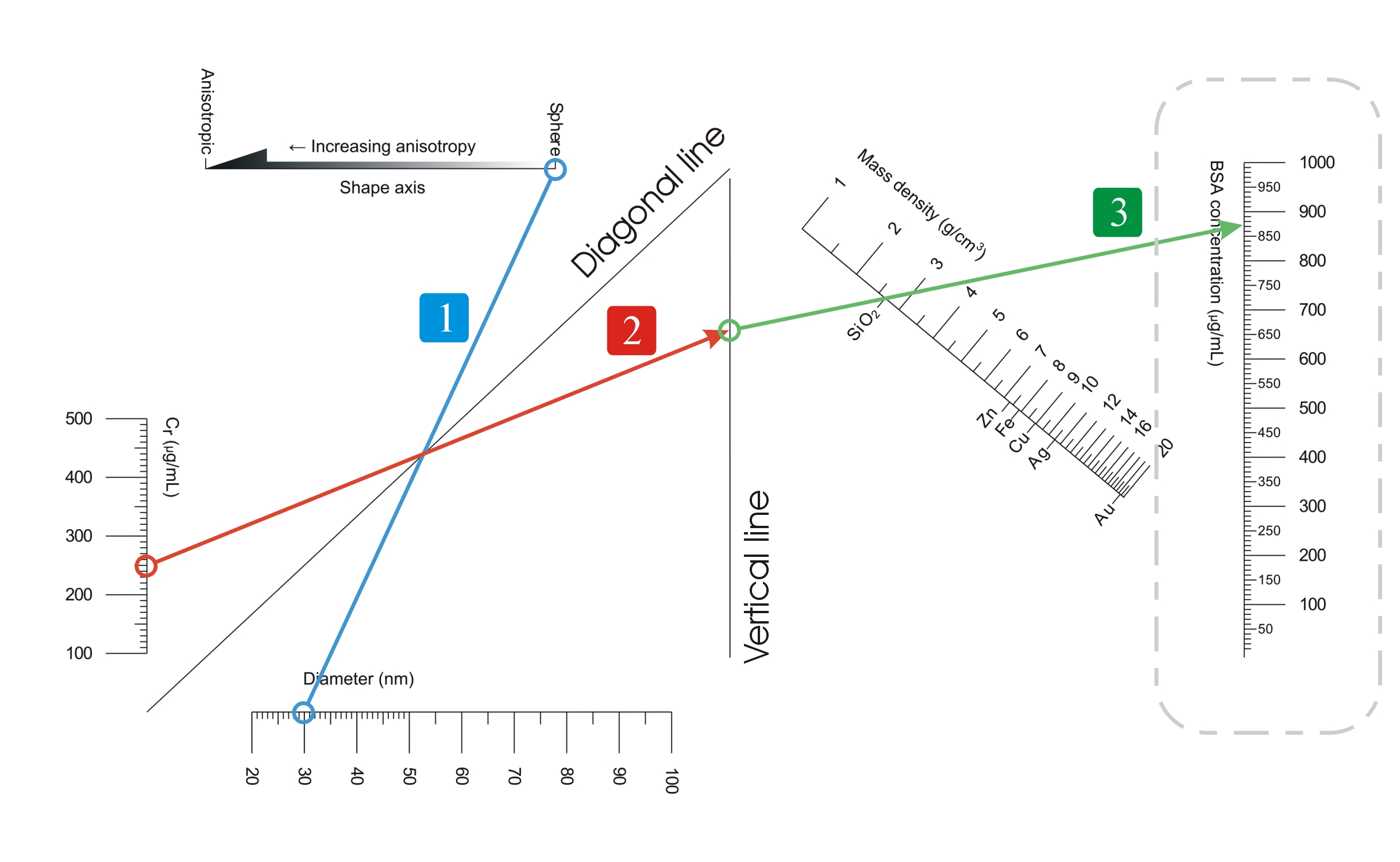


**Figure S4**: The steps of calculating the optimal BSA concentration using the nomograms.

1. First the expected NPs size and shape is considered: draw a straight line between the axis of particle size and the axis of shape (anisotropy and shape factor: ${1\leq S}_{F}\leq3$). The line drawn intersects the diagonal line. For example, if the expected particle is round and has a diameter of 30 nm, the line to be drawn is indicated by blue colour.
2. In the second step the concentration of the suspension is considered. Via the diagonal intersection, draw another line to intersect the vertical line. For example, if the concentration of the NP suspension is 250 $\frac{\mu g}{\mathrm{mL}}$ the line to be drawn is indicated by red colour.
3. Finally, the last line is to be drawn starting from the vertical intersection. This step takes into account the mass density of the NPs (e.g. in case of silica NPs the line is green), and provides the optimal BSA concentration, which is 880µg/mL.


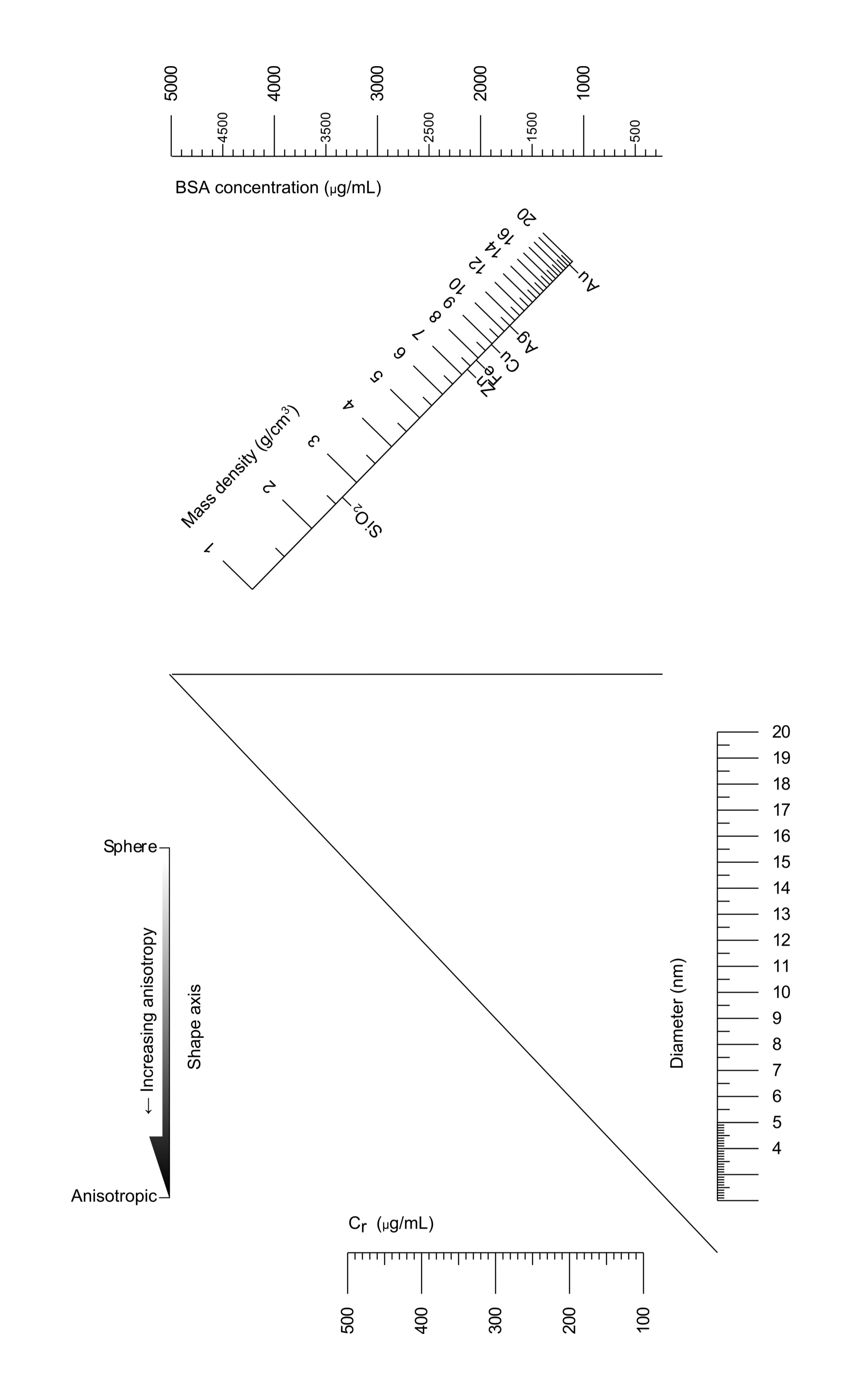

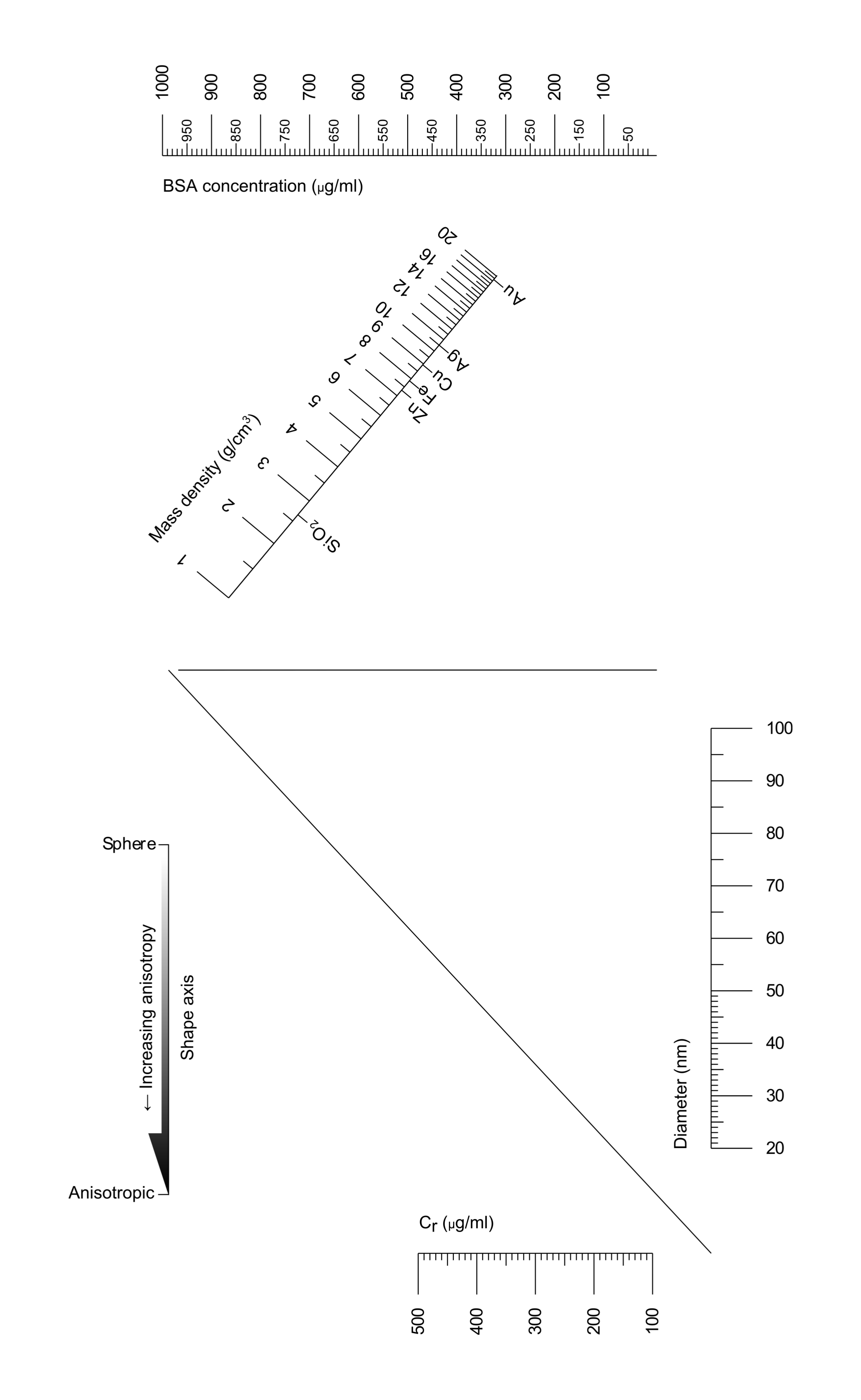


**2. Reconstruction of dynamic light scattering results from TEM micrographs**

The auto-correlation function$c\left( t \right)$ from uniform particles follows a negative exponential as a function of time [^6^](#_ENREF_6):

$c\left( t \right)=ⅇ^{-\left( {}_{R}+{}_{T} \right)t}$ (2.1)

or

$c\left( t \right)=ⅇ^{-{}_{T} t}$ (2.2)

where equation 2.1 and 2.2 correspond to depolarized (DDLS) and polarized scattering (DLS), respectively. ${}_{R}$ and ${}_{T}$ are the relaxation times corresponding to the rotational and translational Brownian motion of the particle. These relaxation times are functions of particle size and morphology. As depicted in Figure S5, TEM analysis resulted in fitting ellipses around the particles, and the three-dimensional object relating to the two-dimensional ellipse is the spheroid, which is obtained by rotating the ellipse about its long axis.


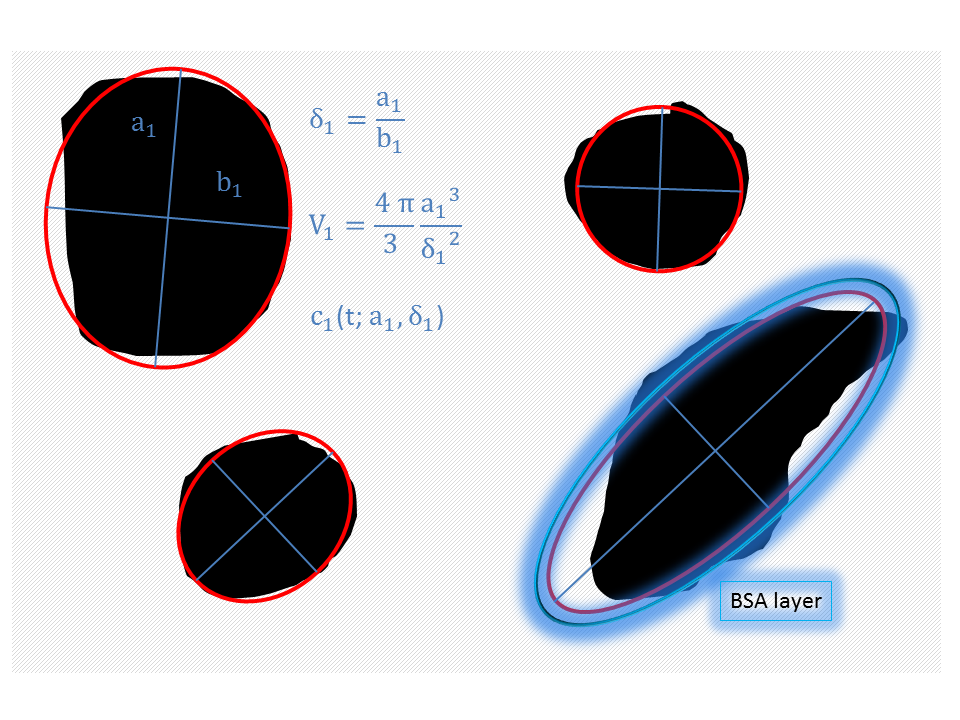


**Figure S5**: The dimensions of each particle observed in TEM are characterized by an ellipsis. The parameters of this ellipsis are used to reconstruct the corresponding intensity-weighted correlation function.

Therefore, we quantify the relaxation times by using spheroids [^7^](#_ENREF_7)^,^[^8^](#_ENREF_8):

${}_{R}\left( a, \delta\right)= \frac{6 k_{B}T}{8\pi}\frac{1}{a^{3}} \frac{3}{2}\frac{\left( 2-\frac{1}{{F\left( \delta\right)}^{2}} \right)F\left( \delta\right)-1}{1-\frac{1}{\delta^{4}}}$ , (2.3)

${}_{T}\left( a, \delta\right)=q^{2}\frac{k_{B}T}{6\pi} \frac{1}{a}F\left( \delta\right)$ , (2.4)

and

$F\left( \delta\right)=\sqrt{\frac{1}{1-\frac{1}{\delta^{2}}}}\mathrm{Ln}\left[ \delta\left( 1+\sqrt{1-\frac{1}{\delta^{2}}} \right) \right]$ $\delta\geq1$, (2.5)

where $a$ is the half of the major axis and $\delta$ is the aspect ratio (Figure SI 1). In the limit of $\delta\to1$, one obtains the expressions corresponding to spherical particles with radius of $a$

${}_{R}\left( a, \delta\to1 \right)=\frac{6 k_{B}T}{8\pi}\frac{1}{a^{3}}$ (2.6)

${}_{T}\left( a, \delta\to1 \right)=q^{2}\frac{k_{B}T}{6\pi}\frac{1}{a}$, (2.7)

where $k_{B}$ is the Boltzmann constant, $T$ the temperature, the viscosity of the solvent, $q$ the momentum transfer $q=\frac{4\pi}{\lambda}n\sin\left( \frac{\theta}{2} \right)$ $,$ $\theta$ the scattering angle, $\lambda$ the wavelength of the laser, and $n$ the refractive index of the solution. Equation 2.1 and 2.2 can be extended for polydisperse particles, by considering that in a given sample each particle contributes to the scattering intensity, depending on its size and anisotropy. The intensity-weighted correlation function can be approximated as

$c\left( t \right)\cong\frac{\sum_{i=1}^{N} {V_{i}}^{2} c_{i}\left( t \right)}{\sum_{i=1}^{N} {V_{i}}^{2}}$ (2.8)

where

$V_{i}=\frac{4 \pi}{3} \frac{{a_{i}}^{3}}{{\delta_{i}}^{2}}$ (2.9)

is the estimated volume of the i^th^ particle, calculated from the best-fitting ellipsis, and $c_{i}\left( t \right)$ is the correlation function corresponding to this particle. An adsorbed BSA layer of 2.5-4 nm thickness [^2^](#_ENREF_2) was taken into account in the reconstructions. To summarize: the reconstruction is based on particles observed and counted in TEM micrographs, and estimates the corresponding correlation function of the suspended state (Figure S6). Considering the very high sensitivity of dynamic light scattering to any deviation that might arise from the incompleteness of the spheroid model we applied in this study, the overall agreement between TEM analysis and dynamic light scattering is outstanding.


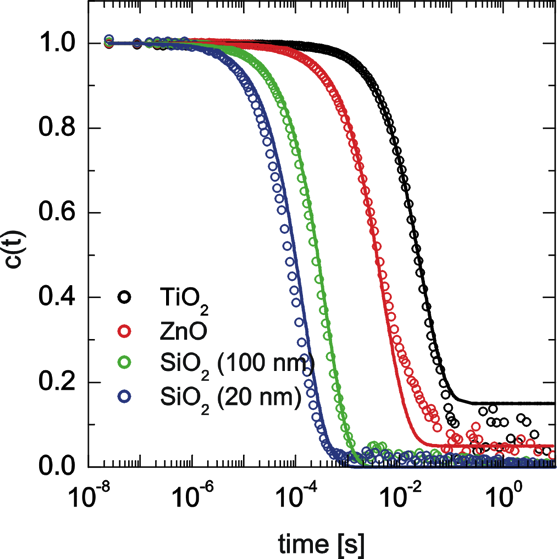


**Figure S6**: Dynamic light scattering spectra (empty symbols) of powder samples and silica NPs compared to the reconstructions (solid lines) based on the TEM analysis of BSA-prepared samples shown on Figure 4. We were unable to obtain a neat dynamic light scattering spectrum from either the Cu or t-CNC particles.

**3. Influence of the grid structure on particle deposition**

At high-curvature surfaces the accumulations of NPs could be observed, e.g. at corners and edges of the TEM grid. Particles deposited onto the copper mesh cannot be imaged by TEM and thus the TEM grids were analysed by scanning electron micrograph (SEM) to visualize silica NPs of 100nm. (Figure S7).


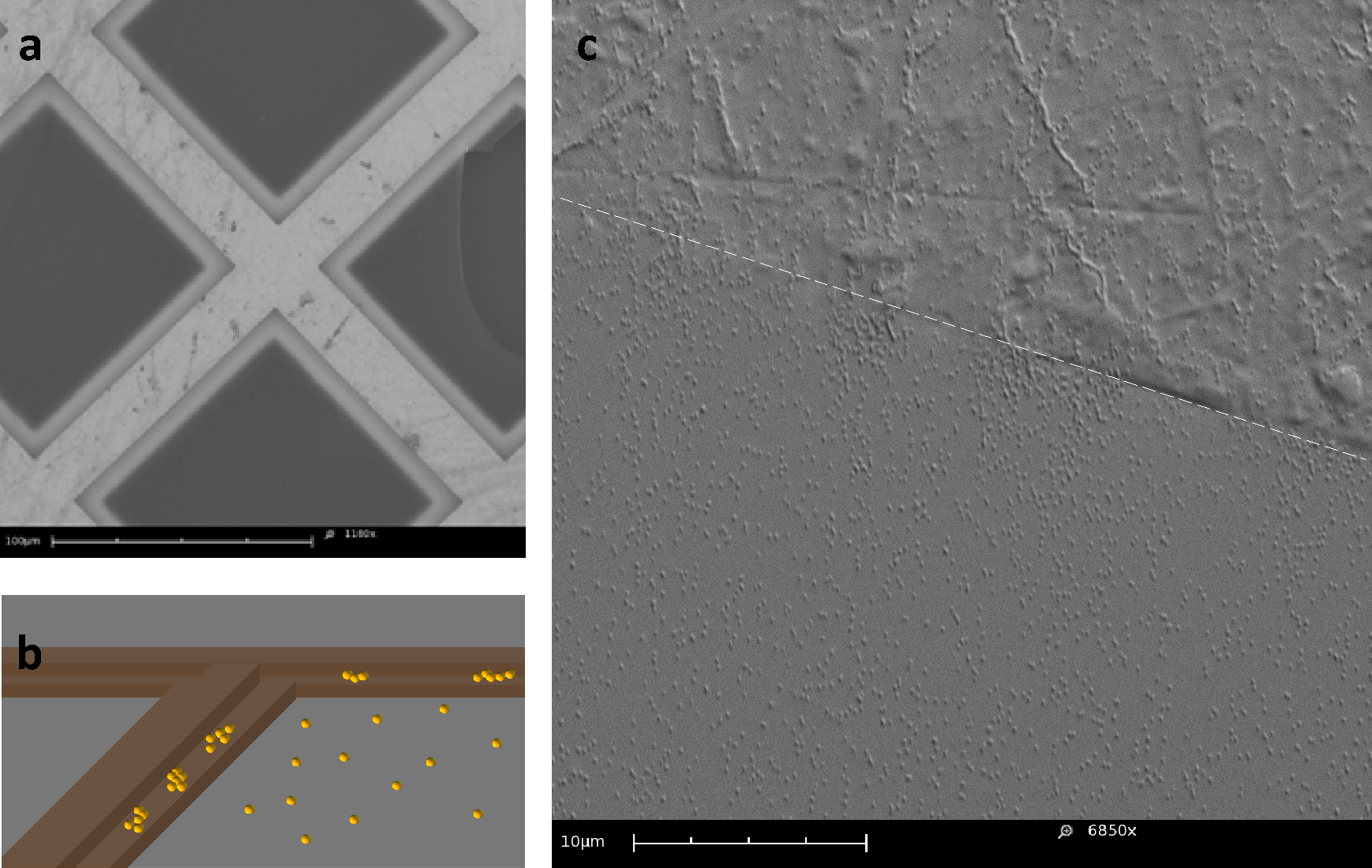


**Figure S7**: (a) SEM micrograph of a TEM grid. (b) Illustration of the grid copper mesh having a profile similar to an inverted T. NPs accumulate near to the corners and edges of this profile. (c) SEM micrograph of silica NPs of 100 nm accumulated near to an edge of the TEM grid.

For further confirmation of the influence of surface curvature on particle deposition from a drying drop, a freshly cleaved mica sheet was used. The surface of the mica sheet is hydrophobic, and TEM grids are frequently coated with a hydrophobic polymer film such as poly(vinyl formal) resin. The deposition of 80 nm gold NPs, observed in SEM, was found to be without any accumulation. The same procedure was repeated, with another freshly cleaved mica sheet where the surface was carefully scratched. As expected, the accumulation of particles near the narrow cut was observed (Figure S8).


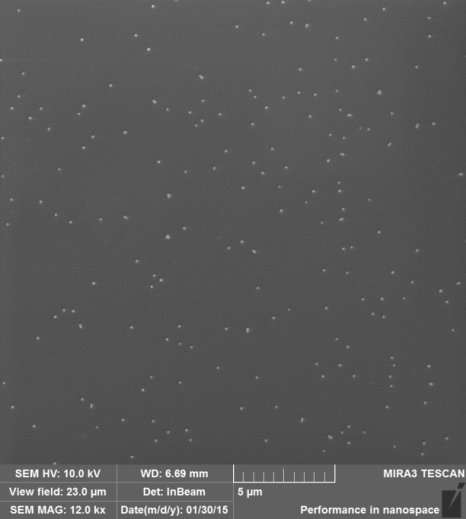

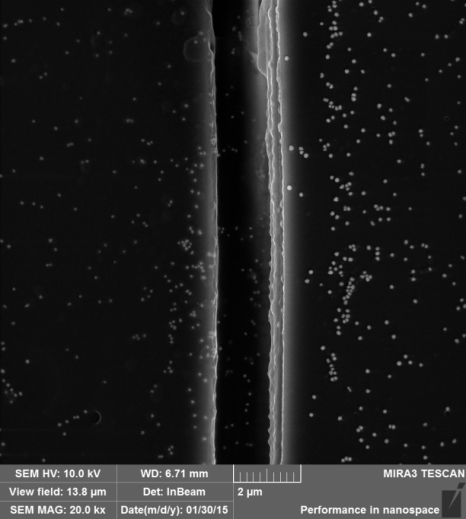


**Figure S8**: Left: SEM micrograph of Au NPs of 80 nm deposited onto a freshly cleaved mica surface. Right: SEM micrograph of the same particles accumulated near to a narrow cut scratched on the surface of a freshly cleaved mica sheet.

**4. Videos of aqueous droplets drying on the TEM grid**

The video “Control.mov” is composed of 361 frames summarizing altogether 36 minutes. It shows a $5 \mu l$ drop of an aqueous suspension of silica particles drop-cast onto a TEM grid and drying under fume hood. Dewetting is evident, and the interface between the grid and the aqueous droplet decreased continuously as drying proceeded. The video “BSA.mov” is composed of 316 frames summarizing altogether 26 minutes. It shows a $5 \mu l$ drop of an aqueous suspension of the very same silica particles as for the control video but this time the sample was prepared via the BSA protocol. The sample then was drop-cast onto a TEM grid and dried under fume hood. Wetting was evident, and the interface between the grid and the aqueous droplet stayed constant over nearly the entire duration of drying.

**References**

1. Brewer, S. H., Glomm, W. R., Johnson, M. C., Knag, M. K. & Franzen, S. Probing BSA binding to citrate-coated gold nanoparticles and surfaces. *Langmuir* **21**, 9303-9307 (2005).

2. Dominguez-Medina, S., McDonough, S., Swanglap, P., Landes, C. F. & Link, S. In Situ Measurement of Bovine Serum Albumin Interaction with Gold Nanospheres. *Langmuir* **28**, 9131-9139 (2012).

3. O'Brien, S. B. G. M. & van den Brule, B. H. A. A. Shape of a small sessile drop and the determination of contact angle. *J. Chem. Soc. Faraday Trans.* **87**, 1579-1583 (1991).

4. Santini, M., Guilizzoni, M. & Fest-Santini, S. X-ray computed microtomography for drop shape analysis and contact angle measurement. *J. Colloid. Interf. Sci.* **409**, 204-210 (2013).

5. Rı́o, O. I. d. & Neumann, A. W. Axisymmetric Drop Shape Analysis: Computational Methods for the Measurement of Interfacial Properties from the Shape and Dimensions of Pendant and Sessile Drops. *J. Colloid. Interf. Sci.* **196**, 136-147 (1997).

6. Pecora, R. *Dynamic Light Scattering: Applications of Photon Correlation Spectroscopy*. (Plenum Press, 1985).

7. Perrin, F. Mouvement brownien d'un ellipsoide - I. Dispersion diélectrique pour des molécules ellipsoidales. *J. Phys. Radium* **5**, 497-511 (1934).

8. Perrin, F. Mouvement Brownien d'un ellipsoide (II). Rotation libre et dépolarisation des fluorescences. Translation et diffusion de molécules ellipsoidales. *J. Phys. Radium* **7**, 1-11 (1936).
